# Antioxidant Functions of the Aryl Hydrocarbon Receptor

**DOI:** 10.1155/2016/7943495

**Published:** 2016-10-18

**Authors:** Cornelia Dietrich

**Affiliations:** Institute of Toxicology, University Medical Center of the Johannes Gutenberg University Mainz, Obere Zahlbacherstr. 67, 55131 Mainz, Germany

## Abstract

The aryl hydrocarbon receptor (AhR) is a transcription factor belonging to the basic helix-loop-helix/PER-ARNT-SIM family. It is activated by a variety of ligands, such as environmental contaminants like polycyclic aromatic hydrocarbons or dioxins, but also by naturally occurring compounds and endogenous ligands. Binding of the ligand leads to dimerization of the AhR with aryl hydrocarbon receptor nuclear translocator (ARNT) and transcriptional activation of several xenobiotic phase I and phase II metabolizing enzymes. It is generally accepted that the toxic responses of polycyclic aromatic hydrocarbons, dioxins, and structurally related compounds are mediated by activation of the AhR. A multitude of studies indicate that the AhR operates beyond xenobiotic metabolism and exerts pleiotropic functions. Increasing evidence points to a protective role of the AhR against carcinogenesis and oxidative stress. Herein, I will highlight data demonstrating a causal role of the AhR in the antioxidant response and present novel findings on potential AhR-mediated antioxidative mechanisms.

## 1. Introduction

The AhR is a transcription factor belonging to the basic helix-loop-helix/PER-ARNT-SIM family [1]. Among this group of proteins, the AhR is the only one that is activated by a ligand. The unliganded receptor is predominantly localized in the cytosol and is associated with two heat shock proteins 90, the immunophilin homologous AhR interacting protein (AIP, also known as ARA9 or XAP2), and the cochaperone p23. After ligand binding, the complex is disrupted which leads to nuclear translocation of the AhR. After heterodimerization with aryl hydrocarbon receptor nuclear translocator (ARNT), the AhR/ARNT heterodimer binds to specific enhancer sequences, known as xenobiotic responsive elements (XREs) or dioxin responsive elements (DREs). Consequently, transactivation of several genes is induced. These genes encode phase I and II xenobiotic metabolizing enzymes, such as* cytochrome P450 monooxygenases (CYP1A1*,* CYP1A2*, and* CYP1B1)* and* glutathione-S-transferases (GSTs)*,* NADPH/quinone oxidoreductase (NQO1)*, and* aldehyde dehydrogenase 3*, respectively (for reviews, see [2, 3]). This AhR-triggered pathway is referred to as the canonical pathway and mediates xenobiotic metabolism.

The AhR was originally discovered due to its stimulation by a variety of planar aromatic hydrocarbons, such as benzo[*a*]pyrene (B[*a*]P), 2,3,7,8-tetrachlorodibenzo-*p*-dioxin (TCDD), and polychlorinated biphenyls (PCBs). The above-described canonical AhR signaling pathway at least partially explains the carcinogenicity of polycyclic aromatic hydrocarbons which are not only detoxified, but also metabolized at the same time to genotoxic compounds. However, it does not help to clarify the molecular mechanisms of toxic effects induced by nongenotoxic AhR ligands, such as TCDD, which is not metabolized.* In vivo* studies in two genetically different rat strains indicate that AhR-driven CYP1A1 induction and tumor promotion can be uncoupled from each other supporting the idea of additional AhR-triggered pathways [4]. To date, several novel noncanonical AhR-driven pathways have been identified and studies in AhR^−/−^ mice provide strong evidence for AhR functions beyond xenobiotic metabolism [4–6]. Alternative binding regions for the AhR or the AhR/ARNT heterodimer have been identified [7–12]. It was also found that the transcription factor NF-*κ*B modulates AhR signaling [13]. In addition to its well-known function as a transcription factor, the AhR has been shown to possess E3 ubiquitin ligase activity [14].

Interestingly, also naturally occurring compounds, such as indoles and several flavonoids (e.g., quercetin), which are present in food, may act as AhR agonists. In search for potential endogenous AhR ligands, diverse compounds such as tryptophan derivatives, arachidonic acid metabolites, equilenin, heme metabolites, and indigoids have been characterized [15]. Also, pharmaceutical drugs may act as AhR ligands, for example, omeprazole or ketoconazole [16, 17]. Furthermore, the AhR is activated by UV photoproducts of tryptophan and regulated by nonligand signals such as cAMP [18, 19]. However, the physiological or toxicological consequences of AhR activation by these ligands are mostly unclear.

Strong evidence indicates that activation of the AhR leads to oxidative stress. This may happen due to metabolism of the ligand and by induction of CYP1 enzymes. It is known that the B[*a*]P-metabolite B[*a*]P-7,8-dihydrodiol is metabolized by aldo-keto reductases forming B[*a*]P-7,8-diol. The catechol groups are sequentially oxidized which results in the formation of a semiquinone radical and B[*a*]P-7,8-dione. Further reduction by NADPH-mediated mechanisms causes again formation of B[*a*]P-7,8-diol. This redox cycling of the B[*a*]P metabolite B[*a*]P-7,8-diol leads to the release of superoxide anions and H_2_O_2_ thereby rapidly inducing oxidative DNA damage [20].* In vitro*, production of reactive oxygen species (ROS) can also be explained, among other mechanisms, by the induction of CYP1A1 (and CYP1B1), uncoupling of electron transfer, and hence superoxide release (for review, see [21]). However, there is increasing evidence that the AhR also displays protective functions against oxidative stress. The AhR target genes* GST* and* NQO1* are well-known enzymes playing important roles in the cellular defense against ROS. CYP1A2 protects against ROS formation by scavenging free electrons [22]. Protective functions of the AhR have also been observed* in vivo*. The AhR reduces colon carcinogenesis in the APC^Min/+^ mouse [14]. AhR^−/−^ mice show higher inflammation in the colon in several experimental models [23, 24], and activation of the AhR attenuates skin inflammation induced by imiquimod [25]. Here, I will highlight data suggesting a causal role of the AhR in the antioxidant response. An overview of the potential mechanisms will be presented and future directions will be proposed. Anti-inflammatory mechanisms of the AhR which also lead to reduction of oxidative stress will not be addressed in detail. Here, the reader is referred to recent excellent reviews [26–28].

## 2. The AhR-Nrf2 Pathway

One of the best studied antioxidant responses mediated by the AhR is activation of nuclear factor erythroid 2 p45-related factor 2 (Nrf2). Nrf2 is a transcription factor which is the key to protection against oxidative stress. It regulates not only a variety of antioxidant enzymes, such as NAD(P)H:quinone oxidoreductase (NQO1), *γ*-glutamylcysteine synthetase, thioredoxin, or heme oxygenase-1, but also several phase I and phase II drug metabolizing enzymes, for example, UDP-glucuronosyltransferase 1A6 (UGT1A6) and glutathione S-transferase (GSTA1/2) as well as multidrug resistance-associated protein transporters (for reviews, see [29, 30]). Activity of Nrf2 is regulated by various mechanisms. Under physiological conditions, in the cytosol, Nrf2 is bound to Kelch-like ECH-associated protein-1 (Keap1), an adaptor protein for the Cullin 3-based ubiquitin E3 ligase complex (Cul3) which mediates ubiquitination of Nrf2, thereby leading to its proteasomal degradation. Hence, the basal levels of Nrf2 are kept constantly low under normoxic conditions. An increase in oxidative/electrophilic stress leads to oxidation of the cysteine residues in Keap1 and, at the same time, inhibition of the activity of the E3 ubiquitin ligase. As a result, Nrf2 protein accumulates, dissociates from Keap1, and is translocated to the nucleus where it dimerizes with Maf or c-Jun. The heterodimer then binds to the so-called antioxidant elements (AREs), also sometimes referred to as electrophile response elements (EpREs), in the promoter region of responsive genes [30, 31]. Activity of Nrf2 can further be regulated by phosphorylation, for example, by protein kinase C (PKC) or the mitogen-activated protein kinases ERK1/2 [32–34]. In addition, expression of Nrf2 is regulated epigenetically by methylation of CpG islands in the promoter and acetylation of histones and microRNA (for review, see [35]) and at the transcriptional level, for instance, in response to oncogenic activation of Ras via a TPA-responsive element (TRE) (for review, see [36]).

Interestingly, there is an overlap between Nrf2 and AhR target genes, that is,* NQO1*,* GSTA2*, and* UGT1A6*. The promoters of these genes contain functional XREs and AREs and, as a consequence, induction of these genes requires activation of AhR and Nrf2 [37] (for review, see [29]).

**Figure 1:**
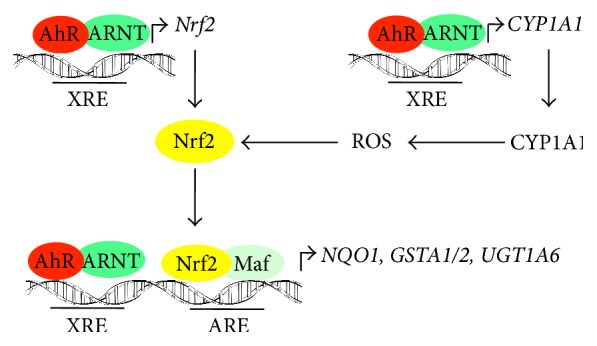
The proposed model for coordinate induction of Nrf2-dependent genes by AhR and Nrf2. Activation of the AhR leads to dimerization with ARNT and transcriptional activation of both Nrf2 and CYP1A1. CYP1A1 increases intracellular ROS thereby stabilizing Nrf2 protein. Nrf2, in association with Maf, binds to AREs, and the AhR/ARNT complex binds to XREs in the promoter regions of* NQO1*,* GSTA1/2*, or* UGT1A6*.

Two different mechanisms probably account for activation of Nrf2 by AhR, that is, (i) direct transcriptional activation of Nrf2 and (ii) generation of ROS by induction of CYP1A1 (Figure 1). Miao and coworkers were the first to show that transcription of Nrf2 is directly regulated by AhR [38]. Exposure of hepatoma cells to TCDD led to induction of Nrf2 mRNA and protein, which was abolished by siRNA, targeted against AhR. They further identified three potential XREs in the murine Nrf2 promoter which appeared to be functional as detected by site-directed mutagenesis and electrophoretic mobility shift assays. Direct binding of the AhR to these XREs was finally shown by chromatin immunoprecipitation assay. It is worth noting that 5 XRE-like elements are found in the human Nrf2 promoter (for review, see [39]). The results of the* in vitro* studies were confirmed* in vivo*. Yeager and coworkers demonstrated that TCCD mediates induction of Nrf2 and its nuclear translocation and transactivation of the Nrf2 target genes* NQO1*,* UGTs*, and* GSTs* in mouse liver. Upregulation of Nrf2 was completely blocked in the liver of AhR^−/−^ mice. Induction of NQO1 was absent in both AhR^−/−^ and Nrf2^−/−^ mice. Furthermore, TCDD-mediated increase in UGT1A6 and several GST isoforms was abolished in Nrf2^−/−^ mice [40, 41]. Similar results concerning NQO1 upregulation have been obtained in Nrf2^−/−^ mice after exposure to the AhR ligand 3-methylcholanthrene (3-MC) [42]. This implies that both AhR and Nrf2 are indispensable for TCDD-mediated NQO1 induction as well as for upregulation of UGT1A6 and GSTA1.

The second explanation for AhR-mediated activation of Nrf2 is based on the observation that activation of the AhR may increase intracellular ROS levels (for review, see [21, 43]). As stated above, production of ROS can be explained* in vitro* by AhR-mediated induction of CYP1A1 (for review, see [21]). An increase in intracellular ROS should lead to both oxidation of Keap1 and release of Nrf2 from the complex.

To date, it has not been finally proven which of the two mechanisms of Nrf2 activation in response to AhR stimulation predominates. Data exist supporting both pathways. Possibly, differences between rodents and humans help explain the divergent results. In mice, TCDD-triggered upregulation of the Nrf2 target gene* NQO1 *is similar in wild-type (wt) and Cyp1a1^−/−^ mouse liver [44]. In line with this observation, TCDD increases expression of Nqo1, Gsta1, and Ugt1a6 mRNA in the livers of Cyp1a1/Cyp1a2/Cyp1b1 triple-null mice [45]. In contrast, CYP1A1 is required for NQO1 induction in human hepatoma cells [46]. TCDD-triggered induction of NQO1 mRNA expression is abolished after transfection of an inactive CYP1A1 mutant and in the presence of the antioxidant N-acetyl cysteine. Oppositely, overexpression of CYP1A1 leads to a similar increase in hydrogen peroxide formation and subsequent NQO1 mRNA compared to TCDD treatment. Hence, the data speak against a pivotal role of CYP1A1 in Nrf2 activation at least in mouse and favour direct transcriptional activation of Nrf2 by the AhR whereas CYP1A1 activation seems to play a role in Nrf2 activation in human liver cells.

In addition to species or cell type specificity, which are well-known characteristics of AhR function, ligand-specific effects have been described recently in human keratinocytes. Ketoconazole, a widely used antifungal compound, leads to AhR/ARNT-dependent induction of Nrf2 protein and its nuclear translocation and induction of NQO1 protein [17]. Importantly, this results in profound inhibition of intracellular ROS generation induced by tumor necrosis factor *α* (TNF*α*) or B[*a*]P. (Interestingly, B[*a*]P leads to an increase in Nrf2 protein, but not to its nuclear translocation.) Ketoconazole itself does not lead to ROS production. It is worthy of note that ketoconazole has only a weak effect on the induction of CYP1A1, thereby supporting the hypothesis that AhR-dependent pathways might be separated from each other in a ligand-dependent way. Similar results were obtained by Takei and coworkers using cynaropicrin, a phytochemical derived from artichoke [47]. Cynaropicrin results in AhR-dependent NRF2 induction, followed by an increased expression of NQO1, and thereby inhibits ROS production mediated by B[*a*]P or UVB, while CYP1A1 mRNA showed a weak increase. Extracts of* Opuntia ficus-indica* also lead to an albeit weak but nonetheless significant increase in Nrf2 activity and NQO1 expression AhR-dependently and reduction of B[*a*]P- or TNF*α*-mediated ROS generation [48].

In summary, these studies show prominent antioxidant functions of the AhR by inducing the Nrf2 response with subsequent upregulation of NQO1, GSTA1/2, and/or UGT1A6. NQO1 has an important function in the reduction of quinones to quinols by a one-step 2e^−^-reduction process, thereby bypassing the semiquinone step and avoiding the generation of ROS. It also maintains endogenous antioxidants in their reduced form, such as coenzyme Q (ubiquinone) and *α*-tocopherol-quinone, reduces lipid peroxidation, and quenches superoxide (for review, see [39, 49]). GSTs are required for detoxification of electrophilic compounds by reaction with glutathione [50]. UGTs also contribute to the antioxidant response by catalyzing conjugation of glucuronic acid, for instance, with quinols, thereby facilitating their excretion [51]. Interestingly, some ligands are able to differentially activate the AhR/Nrf2/NQO1 pathway while the AhR/CYP1A1 axis is only weakly induced.

## 3. Expression of Superoxide Dismutase

Superoxide dismutase (SOD) is a key enzyme in the protection of cells against the harmful superoxide anion radical which constitutively derives from leakage of the mitochondrial respiratory chain. SOD functions by dismutating the superoxide anion radical to molecular oxygen and hydrogen peroxide, the latter being detoxified by catalase. Three isoforms exist in humans: SOD1 (CuZn-SOD), a cytosolic enzyme containing copper and zinc ions in the active site, SOD2 (Mn-SOD), a mitochondrial enzyme bearing a manganese ion, and SOD3, a secreted isoform expressed only by a few cell types (for reviews, see [52, 53]). Whereas to date no XRE has been found in the* SOD2 *promoter, functional XREs have been identified in the promoters of human and rat* SOD1* gene. They were originally identified by deletion/mutation analysis of promoter constructs of rat or human SOD1. Electrophoretic mobility shift assays revealed binding of a TCDD-inducible receptor complex to the XRE. Finally, endogenous SOD1 expression could be stimulated by TCDD in a human hepatoma cell line [54, 55]. The authors later showed that the promoter of* SOD1* also contains a functional ARE and that TCDD-dependent activation of* SOD1* requires both regulatory elements, that is, XRE and ARE [56]. Interestingly, basal expression of SOD1 (and SOD2) was diminished in primary lung fibroblasts derived from AhR^−/−^-mice, but expression of SOD1 could not be increased by cigarette smoke extract in wt-fibroblasts [57, 58]. Very recently, it was demonstrated that fetal pulmonary cells derived from AhR^−/−^-mice displayed reduced SOD1 induction in response to hyperoxia [59].* In vivo*, data concerning induction of SOD1 by AhR ligands are likewise inconsistent. Acute exposure of 3-MC induced upregulation of SOD1 mRNA in mouse liver [60], but not in extrahepatic tissues, such as lung, kidney, or heart tissue [61]. In contrast, no increased expression of SOD1 mRNA could be detected in mouse liver after TCDD treatment [41].

## 4. Nrf2-Independent Antioxidant Functions 

Some* Brassica*-derived phytochemicals exert antioxidant functions. A prominent example is sulforaphane, a potent inducer of Nrf2.* Brassica* vegetables are rich in glucosinolates which are hydrolyzed during digestion to various products including isothiocyanates, thiocyanates, and indoles (for review, see [62]). The isothiocyanate sulforaphane is the hydrolysis product of the glucosinolate glucoraphanin, while indole-3-carbinol (I3C) is a major autolysis product derived from glucobrassicin. Indole-3-carbinol is further converted to various condensation products at acidic pH* in vivo* and* in vitro*, such as 3,3′-diindolylmethane (DIM) and indolo[3,2-*b*]carbazole (ICZ) [63, 64] (for review, see [15]). Interestingly, both ICZ and DIM are potent ligands of the AhR [63] (for review, see [15]). First evidence for a protective function of ICZ against oxidative DNA damage was provided by Bonnesen and coworkers. In the colon carcinoma cell line LS-174, pretreatment with sulforaphane together with ICZ was shown to reduce the level of DNA single-strand breaks in response to B[*a*]P or hydrogen peroxide [65]. Since both B[*a*]P and hydrogen peroxide lead to an increase in intracellular ROS formation [20, 66] (and our own unpublished data), the data suggest an antioxidant function of ICZ. We deeply investigated a potential antioxidant effect of ICZ and revealed that ICZ protects against oxidative DNA damage in various cell lines, including the colon carcinoma cell line Caco-2 [67]. ICZ decreased DNA single-strand breaks (SSB) and 8-oxo-2′-deoxyguanosine (8-oxo-dG) formation induced by hydrogen peroxide, tertiary-butyl-hydroperoxide (t-BOOH), or B[*a*]P when preincubated for 24 h. We found that intracellular ROS levels were attenuated following t-BOOH exposure. Simultaneous addition of ICZ did not protect against t-BOOH-induced SSB formation, nor could we detect a direct radical scavenging effect of ICZ as confirmed by an* in vitro* DPPH assay. Functional inhibition of the AhR and AhR/ARNT defective cell lines demonstrated that the AhR/ARNT pathway is mandatory for the observed ROS defense caused by ICZ, suggesting that AhR-mediated regulation of defense genes is involved. Protection was also detected in response to TCDD. The effect of additional AhR ligands has not been investigated yet. The downstream target(s) of the AhR/ARNT pathway mediating the protection against oxidative stress is not known yet. The observations that (i) the protective effect could not be reversed by trigonelline, an inhibitor of Nrf2, and that (ii) we could detect neither upregulation nor nuclear accumulation of Nrf2 protein speak against involvement of Nrf2. Furthermore, we did not find any increase in SOD1 protein expression after ICZ exposure (unpublished observation). Experiments to unravel the molecular mechanism of AhR-mediated protection against oxidative stress are in progress.

## 5. Induction of Paraoxonases (PONs)

The family of paraoxonases (PONs) comprises three enzymes, that is, PON1, PON2, and PON3. PON1 is predominantly found extracellularly in the blood stream where it is associated with HDL (high-density lipoprotein). PON2 and PON3 are intracellular proteins (for review, see [68]). PON1 and PON3 are synthesized in the liver, and PON2 is ubiquitously expressed in many tissues. Although the precise mechanisms of function are largely unknown, all PONs exert antioxidant functions. PON1, originally identified as a plasma hydrolase metabolizing paraoxon, has important antioxidant properties which, at least partially, account for the protective functions of HDL. For instance, PON1 decreases lipid peroxidation and generation of malondialdehyde. Malondialdehyde is known to trigger intracellular pathways which inhibit endothelial NO-synthase (eNOS) signaling and eNOS-dependent NO production. Hence, proper PON1 function is crucial for NO formation. In addition, PON1 inhibits myeloperoxidase activity in HDLs under inflammatory conditions (for review, see [68]). PON2 and PON3 also attenuate lipid peroxidation by lowering intracellular ROS, especially by maintaining proper mitochondrial function. For instance, PON2 is localized in the inner mitochondrial membrane where it is essential for correct function of the electron transport chain. As a result, PON2 decreases the release of mitochondria-derived superoxide (for reviews, see [68, 69]).

More than ten years ago, Barouki's lab revealed that activation of the AhR leads to induction of PON1 in human hepatoma cells as well as* in vivo* in mouse liver. Interestingly, 3-MC and the phytocompounds quercetin and flavone were strong inducers of PON1 whereas TCDD elicited only a marginal effect on PON1 expression [70]. The fact that induction of CYP1A1 was intense after TCDD, despite being weak after quercetin treatment, indicates again that separate stimulation of AhR pathways is feasible and probably dependent on the ligand. Functional inhibition of the AhR, either pharmacologically or by siRNA, decreased quercetin-triggered PON1 induction, whereas overexpression of the AhR enhanced it. These observations strongly indicate that quercetin-mediated PON1 activation requires AhR. However, PON1 gene expression was not mediated by a classical XRE, but rather by a noncanonical XRE (identified core sequence GCGGG) in the PON1 promoter [70]. Interestingly, resveratrol, which was originally described as an AhR antagonist [71], also led to PON1 expression in an AhR-dependent manner [72]. However, the functional consequence of AhR-mediated induction of PON1* in vivo* has not been studied yet. Recently, Shen and coworkers demonstrated that the dioxin-like PCB126 leads not only to an increase in PON1 mRNA and activity in rat liver, but also to elevation of PON2 and PON3 [73, 74]. Importantly, elevation of PON1 activity could also be detected in the serum. No induction of any PON enzyme could be seen in the lungs of the animals after PCB126 treatment. In contrast, 3-MC upregulated PON3 expression in liver and lung, while TCDD only induced PON3 mRNA in the lung. The underlying mechanism for these ligand-specific effects on PON expression has not been clarified so far. Although the authors did not investigate a causal role of the AhR in PCB126-mediated induction of PON1 (or PON2/3) in the rat, involvement of the AhR is very likely due to the well-known action of planar PCBs on the AhR [75]. In line, non-dioxin-like PCBs did not lead to an increase, but rather to a decrease in serum PON1 activity. Unfortunately, it was not possible to analyze a potential antioxidant effect of PCB126-mediated PON induction, since the PCBs themselves generated oxidative stress [73, 74].

## 6. Upregulation of Sulfiredoxin

One important function of sulfiredoxin (Srxn) is the regeneration of oxidized peroxiredoxins. Peroxiredoxins are known to reduce peroxides which results in the formation of the hyperoxidized, cysteine-sulfinic acid form of peroxiredoxin (Prx-SH→Prx-SO_2_H). Due to its sulfinic acid reductase activity, Srxn reverses hyperoxidation of peroxiredoxin in an ATP-dependent manner (for review, see [76]). Although it is known that transcriptional induction of murine Srxn requires Nrf2 and activator protein-1 (AP-1), Sarill and coworkers recently found that cigarette smoke extract upregulates Srxn mRNA and this depends partially on AhR function. Cigarette smoke extract-mediated Srxn induction was significantly reduced in AhR^−/−^ fibroblasts. Interestingly, cigarette smoke extract induced similar induction of Srxn mRNA in fibroblasts derived from AhR^DBD/DBD^-mice, which carry an AhR mutant unable to bind to XREs [77], compared to wt-mice [58]. This indicates that Srxn upregulation, in response to cigarette smoke extract, does not involve the classical AhR-XRE pathway but rather is mediated by a noncanonical AhR-dependent mechanism. One possible explanation is heterodimerization with the NF-*κ*B protein RelB and binding of the AhR/RelB complex to a promoter region different from the XRE [78]. However, other possible target genes have to be considered since several alternative binding regions for the AhR or the AhR/ARNT heterodimer have been identified [7–12]. Transcriptional activation could also be mediated indirectly by AhR-triggered upregulation of components of the AP-1 family of transcription factors, such as c-Jun or JunD [79–81]. Furthermore, it has been demonstrated that the AhR regulates endogenous levels of miRNAs which could account for increased stabilization of Srxn mRNA [82].

## 7. Protective Function of AhR in Hyperoxic Lung Injury

Although prooxidant functions of the AhR in hyperoxic lung injury have been described [83], other reports demonstrate a protective function of the AhR. Hyperoxia (>95% O_2_) leads to induction of CYP1A1, NQO1, and GSTs* in vitro* and* in vivo* which is considered to require the AhR [59, 84, 85]. In contrast, hyperoxia-induced upregulation of CYP1A2 does not involve AhR signaling [84]. In line with these observations, disruption of AhR function increases ROS generation in fetal primary lung cells in response to hyperoxia and results in higher susceptibility to hyperoxic lung injury in adult and newborn mice [59, 84, 85]. Jiang and coworkers demonstrated an increase in pulmonary edema and neutrophil recruitment after hyperoxic treatment in AhR^−/−^ mice [84]. Although induction of NQO1 and GSTs may partly contribute to the protective function of the AhR, several studies suggest a protective role of CYP1A enzymes. About thirty years ago, Mansour and coworkers revealed that pretreatment of rats or mice with either *β*-naphthoflavone (*β*-NF) or 3-MC decreased hyperoxia-mediated lung toxicity as assessed by pulmonary edema, lipid peroxidation, and lethality [86, 87]. It was later shown that the increase in CYP1A1 activity is correlated with the protection against hyperoxic lung injury in rats which was detected by the amount of pleural effusions [88]. Oppositely, inhibition of CYP1A isoforms by 1-aminobenzotriazole led to increased susceptibility to hyperoxic lung injury and lethality in rats [89] suggesting a protective function of CYP1A enzyme(s). The data were confirmed by studies in Cyp1a1^−/−^ mice. Cyp1a1^−/−^ mice were more sensitive towards hyperoxia-mediated pulmonary injury; they showed increased neutrophil infiltration and higher amounts of lipid peroxidation [90]. Noteworthy were the higher levels of F_2_-isoprostanes (and isofurans) in the lungs of Cyp1a1^−/−^ compared to wt-mice and this provides an explanation for the protective function of CYP1A1. F_2_-isoprostanes derive from nonenzymatic peroxidation of fatty acids, predominantly arachidonic acid, thereby forming prostaglandin F_2_-like products. F_2_-isoprostanes are prominent markers for oxidative stress* in vivo*. Also, circulating F_2_-isoprostanes are considered to play a role in inflammatory lung diseases by various receptor-triggered pathways (for review, see [91]). In a recent work by Wang and coworkers [92], it was shown that knocking out Cyp1a2, primarily expressed in the liver, also increased susceptibility for hyperoxic lung injury. This was assessed by the ratio weight_lung_/weight_body_ and histology, pulmonary neutrophil infiltration, cytokine expression, lipid peroxidation, and F_2_-isoprostane levels in liver and lung [92]. The authors finally provide evidence for CYP1A2-mediated metabolism of PGF_2_-*α in vitro* supporting the idea that CYP1A1 and CYP1A2 protect against oxidative stress by decreasing the amount of lung- or liver-derived circulating F_2_-isoprostanes. Hence, these data suggest a protective function of CYP1A1 and CYP1A2 against hyperoxic lung injury, maybe due to decreased lipid peroxidation.

Finally, AhR-mediated protection might be due to its interaction with the NF-*κ*B protein RelB. In an* in vitro* approach, Zhang and coworkers used AhR-deficient human fetal pulmonary microvascular endothelial cells (HPMEC) to unravel mechanisms underlying the protective function of the AhR [59]. Downregulation of AhR expression by RNA interference led to increased ROS formation and augmentation of hyperoxia-mediated toxicity. The authors uncovered attenuation of CYP1A1 and NQO1 (and SOD1) expression in AhR-deficient cells and additionally a decrease in nuclear RelB expression. Indeed, several studies suggest that the AhR reduces lung inflammation by upregulating RelB expression [57, 93]. RelB is supposed to be a negative regulator of the proinflammatory NF-*κ*B pathway, possibly by interaction with p50, thereby diminishing the amount of p50 to form active dimers with the p65 protein (p50 : p65), the classical NF-*κ*B complex. In summary, there is evidence for a protective role of the AhR in hyperoxic lung injury which is probably mediated by regulating the expression of antioxidant enzymes, such as NQO1 and CYP1A1/2. Additional mechanisms might also contribute to protection, such as increased expression of RelB leading to inhibition of the proinflammatory NF-*κ*B pathway.

## 8. Concluding Remarks

The presented data clearly indicate that the AhR plays a role in the antioxidant defense. Protection might be mediated by different mechanisms, such as AhR-dependent activation of Nrf2, PONs, SOD1, or CYP1A1/2 or by additional mechanisms which remain to be clarified. Also, noncanonical pathways seem to be involved, such as upregulation of sulfiredoxin which is independent of a classical XRE.

For some, but not for all of the antioxidant mechanisms, the* in vivo* relevance has been demonstrated in animal models. Nrf2^−/−^ mice are prone to increased oxidative stress, inflammation, neurodegeneration, and carcinogenesis (for review, see [94]). AhR^−/−^ mice are more susceptible to colon carcinogenesis, inflammation, and hyperoxic lung injury [84] (for review, see [95]). Lung fibroblasts gained from patients suffering from chronic obstructive pulmonary disease (COPD) express less AhR protein than patients without COPD and show decreased upregulation of NQO1 and Srxn in response to cigarette smoke extract [58]. Low expression of the AhR is also found in inflammatory bowel disease [96]. Oppositely, targeting the Nrf2 pathway by Nrf2-activating compounds, such as sulforaphane, protects against oxidative stress-mediated diseases like carcinogenesis, neurodegeneration, and cardiovascular illnesses in different animal models (for review, see [97]). Hence, it is conceivable that activation of the AhR-Nrf2 signaling pathway by AhR ligands should also exert chemopreventive effects. However, a direct link between AhR activation, Nrf2 induction, inhibition of ROS, and chemoprevention has not been shown* in vivo* yet. It also remains unproven whether AhR-mediated activation of PONs will lead to protection against atherosclerosis and whether ICZ is chemopreventive in animal models. Finally, the mechanism(s) of possible antioxidant functions of CYP1A1/2 remain to be elucidated.

**Figure 2:**
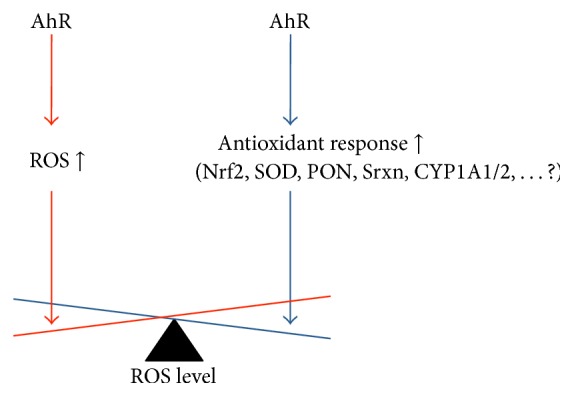
Hypothesis of AhR-mediated regulation of intracellular ROS. Different pathways of the AhR are activated at the same time, one leading to an increase in cellular ROS and the other(s) resulting in an antioxidant response. Possible antioxidant mechanisms are AhR-triggered activation of Nrf2, SOD, PONs, Srxn, CYP1A1/2, or, very likely, other enzymes which remain to be identified. Depending on the cell type, organ, ligand, or additional factors, either the prooxidant or the antioxidant AhR pathway predominates.

The described findings are in contrast to the well-known increase in oxidative stress in response to AhR activation, for instance, induced by TCDD (for reviews, see [21, 43]). It has to be emphasized that the observed effects on DNA damage* in vitro* seem to depend on the cell type tested and are generally quite small [98–100]. Oxidative stress results from the net balance of oxidative and antioxidative mechanisms. Moreover, activation of the AhR will lead to induction of more than one signaling pathway. It is therefore reasonable to hypothesize that oxidant and antioxidant responses are triggered by the AhR in parallel, very likely with different kinetics (Figure 2). Protective mechanisms keeping the level of oxidatively damaged DNA low, despite the generation of oxidative stress, would also explain the lack of TCDD-mediated mutagenicity in rats [101]. In view of the well-known cell type and organ specificity of AhR function, it is plausible to assume that, depending on the cell type or organ, oxidative or antioxidative AhR pathways predominate [67].

From a mechanistic and therapeutical view, it would be worth searching for more nontoxic ligands which selectively activate protective AhR-dependent pathways. As outlined above, discrimination could be observed by using ketoconazole, cynaropicrin, or quercetin. Different effects on AhR signaling were also detected when comparing TCDD and DIM [102]. The reasons for such ligand-specific effects remain unclear. One possible explanation is the recruitment of different cofactors [103]. Identification of selective, ideally nontoxic ligands not only would contribute to specifically triggering protective AhR signaling pathways, but also would probably help to gain a better insight into the mechanisms underlying AhR function.
